# Insertion of Horizontally Transferred Genes within Conserved Syntenic Regions of Yeast Genomes

**DOI:** 10.1371/journal.pone.0006515

**Published:** 2009-08-05

**Authors:** Thomas Rolland, Cécile Neuvéglise, Christine Sacerdot, Bernard Dujon

**Affiliations:** 1 Unité de Génétique Moléculaire des Levures (CNRS URA 2171, UFR927 Université Pierre et Marie Curie), Département Génomes et Génétique, Institut Pasteur, Paris, France; 2 INRA UMR1238, CNRS UMR2585, AgroParisTech, Microbiologie et Génétique Moléculaire, Thiverval-Grignon, France; Universidade de Sao Paulo, Brazil

## Abstract

Horizontal gene transfer has been occasionally mentioned in eukaryotic genomes, but such events appear much less numerous than in prokaryotes, where they play important functional and evolutionary roles. In yeasts, few independent cases have been described, some of which corresponding to major metabolic functions, but no systematic screening of horizontally transferred genes has been attempted so far. Taking advantage of the synteny conservation among five newly sequenced and annotated genomes of *Saccharomycetaceae*, we carried out a systematic search for HGT candidates amidst genes present in only one species within conserved synteny blocks. Out of 255 species-specific genes, we discovered 11 candidates for HGT, based on their similarity with bacterial proteins and on reconstructed phylogenies. This corresponds to a minimum of six transfer events because some horizontally acquired genes appear to rapidly duplicate in yeast genomes (*e.g.* YwqG genes in *Kluyveromyces thermotolerans* and serine recombinase genes of the IS607 family in *Saccharomyces kluyveri*). We show that the resulting copies are submitted to a strong functional selective pressure. The mechanisms of DNA transfer and integration are discussed, in relation with the generally small size of HGT candidates. Our results on a limited set of species expand by 50% the number of previously published HGT cases in hemiascomycetous yeasts, suggesting that this type of event is more frequent than usually thought. Our restrictive method does not exclude the possibility that additional HGT events exist. Actually, ancestral events common to several yeast species must have been overlooked, and the absence of homologs in present databases leaves open the question of the origin of the 244 remaining species-specific genes inserted within conserved synteny blocks.

## Introduction

The transfer of genetic information between organisms normally separated by reproductive barriers, a process now known as horizontal (or lateral) gene transfer (HGT or LGT), was for long time considered limited to specific systems such as, for example, transducing viruses or bacteriophages (reviewed in [1]). With the rapidly increasing number of genome sequences, examples of horizontally transferred genes accumulated, especially for bacterial genomes where they play important functional and evolutionary roles [2], [3]. The role of HGT in eukaryotic evolution was generally regarded as more limited if one excludes their ancestral organelle endosymbioses, but is now gaining greater attention with the increasing number of well supported cases, many of which with significant functional implications [4]. Although the majority of such cases concerns protists with phagotrophic life style [5], significant examples have recently been reported for fungal genomes, especially plant pathogens [6] or species living in complex microbial populations such as rumen [7]. Horizontal transfer has also been proposed for a variety of non-infective selfish genetic elements irregularly found in fungal species, such as plasmids, mycoviruses, mobile group I introns and their encoded homing endonucleases, and even transposons [8]. It is also debated as the possible origin of clusters of genes encoding secondary metabolite enzymes [8]–[10].

Among fungi, hemiascomycetous yeasts represent a homogeneous, monophyletic subdivision in which numerous genomes have been sequenced [11]–[13], including several isolates of *Saccharomyces cerevisiae* [14]–[18 and www.broad.mit.edu], one of the most extensively studied eukaryotic genome. Beside the selfish genetic elements mentioned above, few genes of putative bacterial origin were recognized in the genomes of several hemiascomycetous yeasts such as *Eremothecium (Ashbya) gossypii* [19], *Kluyveromyces lactis*, *Debaryomyces hansenii*, *Yarrowia lipolytica* [20], *S. cerevisiae* [15], [19], [21]–[23], *Dekkera bruxellensis* [24] or *Candida parapsilosis* [25]. Most of them encode metabolic enzymes that may play important physiological roles in the adaptation of the host species. Perhaps the most spectacular case so far is the acquisition of a bacterial gene encoding di-hydroorotate dehydrogenase (possibly from a *Lactococcus*) by an ancestor of all *Saccharomycetaceae*, forming the *URA1* gene encoding the cytoplasmic enzyme active even in anaerobic condition, while the ancestral *URA9* gene encoding the strictly aerobic mitochondrial enzyme was secondarily lost in the *Saccharomyces sensu stricto* and a few other species [22]. Independent transfers of the same bacterial function to distinct eukaryotic clades seem to have occurred repeatedly in yeasts and other organisms, often in replacement of ancestral eukaryotic genes lost during evolution [21], [23], [25].

Despite the well-documented above examples, cases of horizontal gene transfer in yeasts, and in fungi in general, remain anecdotal. A reason for this may be that genes of foreign origin were not systematically sought for in available genome sequences. Another reason is that, despite suggestive signatures such as distinctive nucleotide composition or biased codon usage, the gold standard for identifying HGT remains phylogenetic incongruence of the suspected gene(s) with respect to the accepted species phylogeny. This discriminative criterion transfers the burden of proof to the proper taxonomic sampling of the sequenced species across phylogenies, a problem rarely solved at present. In yeasts for example, extensive genomic studies have focused on *S. cerevisiae* and the human pathogen *C. albicans*, and their close relatives, leaving the broad evolutionary range of other hemiascomycetes relatively unexplored [11], [12]. In a recent work, the genomes of five protoploid species of *Saccharomycetaceae*, belonging to four distinct clades that separated from *S. cerevisiae* before its genome duplication, have been analyzed and compared [13]. This set consists of three newly sequenced genomes, *Zygosaccharomyces rouxii*, *Kluyveromyces (Lachancea) thermotolerans* and *Saccharomyces (Lachancea) kluyveri,* and two previously published ones, *K. lactis* [20] and *E. gossypii* [26]. It is thought to reflect the ancestral genome of the *Saccharomycetaceae* family. Despite their broad evolutionary distances, distinct metabolic properties and habitat, the genomes of these species have numerous conserved blocks of synteny within which individual, lineage-specific gene insertion or loss can be examined. We used this criterion to identify inserted genes among which, after analysis, some proved to represent novel cases of HGT from bacterial origins. The presence of these genes suggests that HGT may be more frequent and functionally important than usually suspected and, consequently, may play a significant role in genome evolution.

## Results

### Conservation of synteny and identification of putative gene transfers

**Figure 1:**
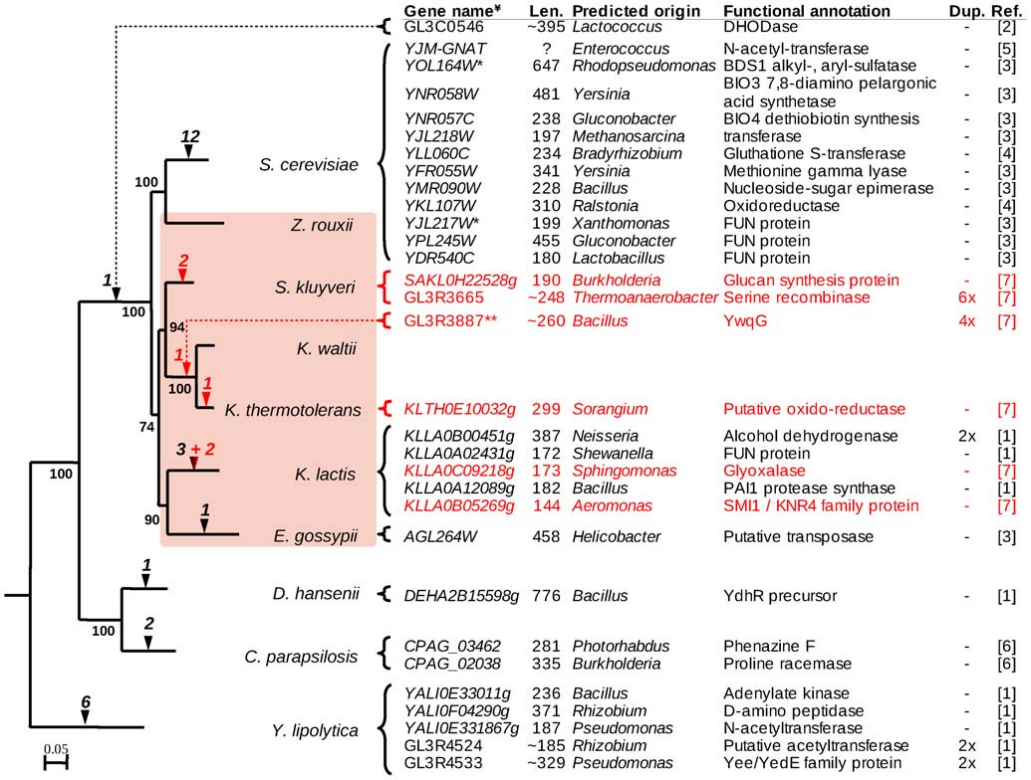
Phylogenetic tree of the *Saccharomycetaceae*, with published HGT cases. The tree was constructed by maximum likelihood using PHYML, from alignments of conserved protein families with only one member per species and corresponding to true orthologs as defined by SONS [13]. Alignments were performed using the MAFFT algorithm and further cleaned with Gblocks before concatenation (53 families, 19144 residues). Bootstrap values are indicated next to the nodes. Triangles represent cases of HGT (black are published cases and red are cases discussed in this paper). References: (1) Dujon *et al.* 2004; (2) Gojkovic *et al.* 2004; (3) Hall *et al.* 2005; (4) Hall and Dietrich, 2007; (5) Wei *et al.* 2007; (6) Fitzpatrick *et al.* 2008; (7) This work. Len.: length in amino-acids (for multigene families, the average is shown). Dup.: Number of copies of transferred gene. FUN: unknown function. ^¥^ In cases of duplication and/or ancestral HGT, the Génolevures families replace individual gene names. * Highly similar orthologs of *S. cerevisiae* genes *YOL164W* and YJL217W have been identified in *K. thermotolerans*, suggesting more ancestral HGT events. ** The gene is present in four copies in *K. thermotolerans*, but only once in *K. waltii*.

**Figure 2:**
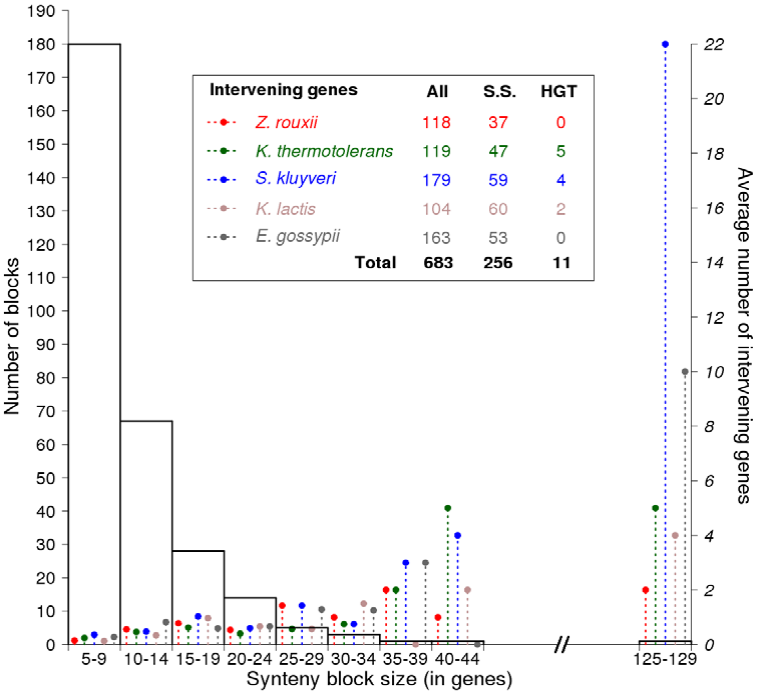
Size distribution of conserved synteny blocks among the five protoploid *Saccharomycetaceae*, and intervening gene numbers. Synteny block common to all 5 protoploid *Saccharomycetaceae* species (inset) were classified by their number of anchoring points (abscissa, Materials and Methods). The number of blocks in each size category is referred to the scale on the left axis. The mean number of intervening genes per species and per block category is represented by dotted lines (scale on the right axis), and total figure for each species are in inset. All: total number of intervening genes. S-S: total number of species-specific intervening genes. HGT: total number of horizontally transferred genes.

Analysis of synteny among five protoploid genomes of the *Saccharomycetaceae* family (a phylogeny of these species among other sequenced hemiascomycetes is illustrated by Figure 1) has revealed a striking conservation of gene order, and most often orientation in the different pairwise comparisons [13]. Synteny blocks of 14 to 26 genes on average, cover over 80% of all protein-coding genes. We extended here the analysis of synteny block conservation to all five genomes simultaneously (Materials and Methods). A total of 300 synteny blocks, ranging in size from 5 to 42 anchor points was found (Figure 2), with the notable exception of a block made of 127 anchor points (This block is discussed in [27]). The number of blocks decreases as their size increase, and only a few blocks with more than 30 anchor points are found. In total, conserved synteny blocks common to all five species cover *ca.* 65% of each genome among which we could examine the insertion of novel genes in one species compared to the others. To do so, we established a list of “intervening genes”, inserted within a block in one of the five species without altering its overall conservation (*i.e.* flanking genes remain unchanged in order and almost always in orientation). They are more numerous in *S. kluyveri* and *E. gossypii*. The number of intervening genes is found roughly proportional to the block size (Figure 2). Out of the 300 synteny blocks examined, we initially found 682 intervening genes. Their translation products were individually compared to all known hemiascomycetous genomes in order to test their uniqueness or find similarities (Materials and Methods). A total of 427 genes have ectopic homologs in the other yeast species and were, therefore, considered as resulting from internal chromosomal rearrangements. Among the remaining 255 species-specific genes, 244 have no significant hit (according to parameters, Materials and Methods), and thus could not be further studied (See Discussion and Supplementary Table S1). The remaining 11 gene products show significant sequence similarity with bacterial proteins and are, therefore, candidates for HGT. The candidates include four single intervening genes (one in *K. thermotolerans*, two in *K. lactis* and one in *S. kluyveri*), four members of a multi-gene family in *K. thermotolerans*, and three members of a multi-gene family in *S. kluyveri*. These HGT candidates, together with other previously published cases in hemiascomycetous yeasts, are summarized in Figure 1.

### Single horizontally acquired genes

**Figure 3:**
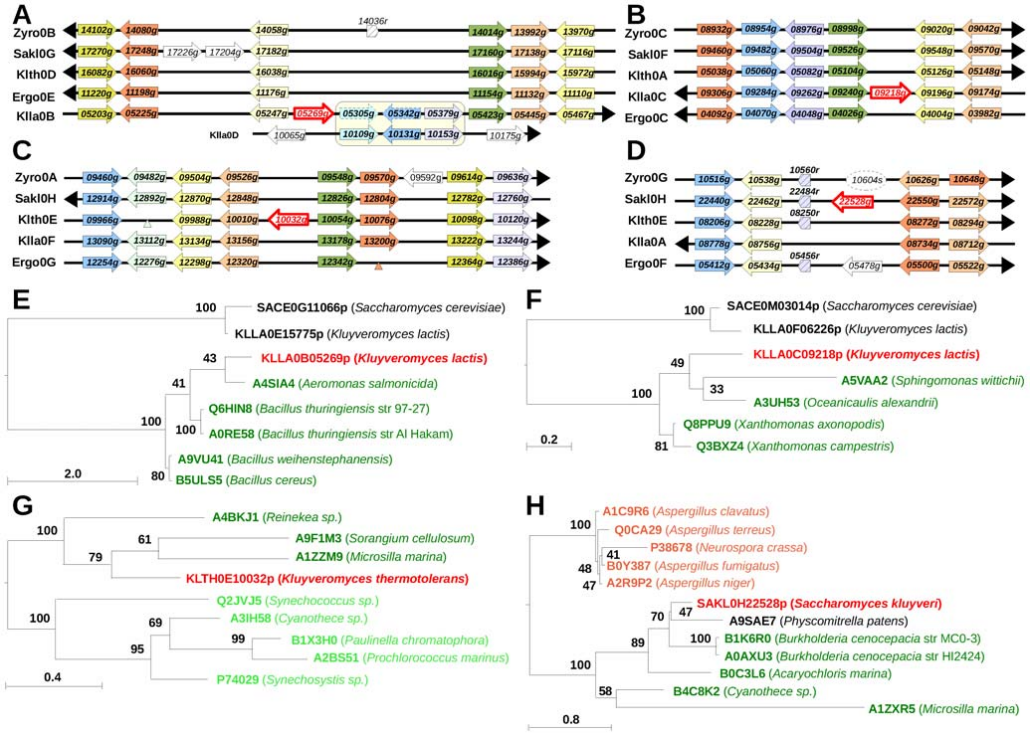
Single HGT candidates in *K. lactis*, *K. thermotolerans* and *S. kluyveri*. (A) Part of the conserved synteny block surrounding the *KLLA0B05269g* gene (previously reported in [20]). Only protein coding and tRNA genes are shown. Orthologous genes are colored, intervening genes are white, tRNA genes are indicated by short hatched arrows, pseudogenes are identified by dotted lines. Arrows represent gene orientation. The segmental duplication between chromosomes B and D of *K. lactis* is shown at the bottom of the figure. *KLLA0D10109g* is similar to *S. cerevisiae YOR120W GCY1*, *KLLA0D10131g* similar to *S. cerevisiae YKL221W MCH2,* and *KLLA0D10153g* and *KLLA0B05379g* are slightly similar to *S. cerevisiae YKL222C* protein of unknown function. (B) Part of the conserved synteny block surrounding the *KLLA0C09218g* gene. Same legend as (A). (C) Part of the conserved synteny block surrounding *KLTH0E10032g* gene. Same legend as (A). Triangles represent single gene species-specific deletions. (D) Part of the conserved synteny block surrounding *SAKL0E22528g*. Same legend as (A). *ZYRO0G10604s* is the centromere of chromosome G in *Z. rouxii* (dotted oval). Note the local inversion of two genes in *Z. rouxii*. (E) Phylogenetic tree reconstructed from sequence alignment of 81 sites of the *KLLA0B05269g* product, bacterial proteins (green) and products of *S. cerevisiae SMI1* gene and its *K. lactis* homolog (*YGR229C*/SACE0G11066p and KLLA0E15775p, in black). Bootstrap values are indicated next to the nodes and branch length scale is shown at bottom left. (F) Phylogenetic tree reconstructed from sequence alignment of 65 sites of the *KLLA0C09218g* product, bacterial proteins (green) and the *S. cerevisiae* glyoxalase I gene (*YML004C*/SACE0M03014p) and its *K. lactis* homolog (KLLA0F06226p). Same legend as (B). (G) Phylogenetic tree reconstructed from sequence alignment of 85 sites of the *KLTH0E10032g* product and bacterial proteins (dark green, cyanobacteria in light green). Same legend as (B). (H) Phylogenetic tree reconstructed from sequence alignment of 72 sites of the *SAKL0H22528g* product, bacterial and moss proteins (green) and *Pezizomycotina* proteins (light red). Same legend as (B).

The *K. lactis KLLA0B05269g* gene, coding a 144 amino-acid long protein, is contained in a conserved synteny block made of 42 anchor points (one of the largest conserved synteny block among our five protoploid *Saccharomycetaceae*, Figure 3A) and shares no similarity with any yeast, fungal or eukaryotic protein presently known. Instead, it shares high sequence similarity with proteins of *Aeromonas salmonicida* (53% amino-acid identity, Table 1) and various *Bacillus* species (including *B. thuringiensis*, *B. cereus*, and *B. weihenstephanensis*, 42–49% identity, see alignment in Supplementary Figure S1A). None of these proteins is annotated, except the *B. cereus* protein annotated as a member of the SMI1/KNR4 family, involved in the regulation of the cell wall synthesis in yeast [28]. The SMI1 protein of *S. cerevisiae* (*YGR229C*) has homologs in all hemiascomycetous yeasts, including *K. lactis* (KLLA0E15775p protein) but those are 3- to 4-fold longer in sequences than *KLLA0B05269g* and share only limited sequence identity with it (average 22%, Table 1). As expected, the tree reconstructed with *S. cerevisiae* and *K. lactis* SMI1 proteins shows that the *KLLA0B05269g* gene product is closer to bacterial proteins than to the yeast SMI1 homologs (Figure 3E), suggesting a recent acquisition of this gene in the *K. lactis* lineage from a bacterium. Its actual function remains to be established. Interestingly, the *KLLA0B05269g* gene is flanked in its distal side by a segmental duplication of 5.5 kb with chromosome D (Figure 3A). This segment involves three genes, two of which being annotated as pseudogenes on chromosome B. The contiguity of the HGT candidate with the segmental duplication (only ∼2 bp) raises the question of a possible link between the two events (Discussion).

**Table 1:** Pairwise sequence identity between single HGT candidate products, best bacterial hits and yeast/fungal similarly annotated proteins.

Query gene	Génolevures/Uniprot annotation	Species	Reference*	Length (aa)	Identity (%)**
***KLLA0B05269g*** ** (144 amino-acids)**	hypothetical protein ASA_0453	*Aeromonas salmonicida*	A4SIA4	143	53
	hypothetical protein BT9727_2262	*Bacillus thuringiensis* str. 97–27	Q6HIN8	142	49
	hypothetical protein BALH_2200	*Bacillus thuringiensis* str. Al Hakam	A0RE58	142	48
	hypothetical protein BcerKBAB4_2135	*Bacillus weihenstephanensis*	A9VU41	143	44
	SMI1/KNR4 family protein BCAH1134_3336	*Bacillus cereus*	B5ULS5	134	42
	SMI1 homolog	*Kluyveromyces thermotolerans*	KLTH0C01760p	516	28
	SMI1 homolog	*Saccharomyces kluyveri*	SAKL0G15466p	515	26
	ADL139W – SMI1 protein	*Eremothecium gossypii*	ERGO0D06072p	657	26
	SMI1 homolog	*Zygosaccharomyces rouxii*	ZYRO0B15180p	619	24
	YGR229C – SMI1 protein	*Saccharomyces cerevisiae*	SACE0G11066p	505	24
	*KLLA0E15862g* a – KNR4/SMI1 family protein	*Kluyveromyces lactis*	KLLA0E15775p	535	23
***KLLA0C09218g*** ** (173 amino-acids)**	hypothetical protein XAC0586	*Xanthomonas axonopodis* pv. Citri str. 306	Q8PPU9	164	44
	putative glyoxalase/bleomycin resistance protein/dioxygenase XCV0638	*Xanthomonas campestris* pv. Vesicatoria str. 85–10	Q3BXZ4	131	41
	hypothetical protein OA2633_07919	*Oceanicaulis alexandrii* HTCC2633	A3UH53	131	39
	glyoxalase/bleomycin resistance protein/dioxygenase Swit_2866	*Sphingomonas wittichii* RW1	A5VAA2	127	39
	Glyoxalase I homolog	*Saccharomyces kluyveri*	SAKL0A02244p	341	25
	KLLA0F06226p	*Kluyveromyces lactis*	KLLA0F06226p	338	24
	YML004C - Glyoxalase I	*Saccharomyces cerevisiae*	SACE0M03014p	326	25
	Glyoxalase I homolog	*Zygosaccharomyces rouxii*	ZYRO0D09064p	347	28
	Glyoxalase I homolog	*Kluyveromyces thermotolerans*	KLTH0A05896p	346	26
***KLTH0E10032g*** ** (299 amino-acids)**	Hypothetical protein Sce1221	*Sorangium cellulosum* So ce56	A9F1M3	292	37
	Putative uncharacterized protein M23134_00952	*Microscilla marina* ATCC 23134	A1ZZM9	277	27
	Putative NADH-ubiquinone oxido-reductase MED297_07641	*Reinekea sp.* MED297	A4BKJ1	284	29
	NAD-dependent epimerase/dehydratase family protein CYA_1049	*Synechococcus sp.* JA-3-3Ab	Q2JVJ5	318	27
	Putative uncharacterized protein CY0110_10507	*Cyanothece sp.* CCY 0110	A3IH58	325	27
	Putative chaperon-like protein for quinone binding in Photosystem II ycf39	*Paulinella chromatophora*	B1X3H0	320	24
	Putative chaperon-like protein for quinone binding in Photosystem II A9601_13281	*Prochlorococcus marinus* AS9601	A2BS51	320	26
	Ycf39 protein	*Synechocystis sp.* PCC 6803	P74029	219	22
***SAKL0H22528g*** ** (190 amino-acids)**	Predicted protein PHYPADRAFT_163804	*Physcomitrella patens*	A9SAE7	248	38
	MoeA domain protein Bcenmc03_4042	*Burkholderia cenocepacia* str MC0-3	B1K6R0	674	38
	MoeA domain protein Bcen2424_3480	*Burkholderia cenocepacia* str HI2424	A0AXU3	693	38
	Putative uncharacterized protein M23134_06735	*Microscilla marina* ATCC 23134	A1ZXR5	194	32
	Protein involved in beta-1-3-glucan synthesis Cyan7425DRAFT_0799	*Cyanothece sp.*	B4C8K2	180	31
	Putative uncharacterized protein AM1_4879	*Acaryochloris marina* str MBIC 11017	B0C3L6	165	31
	1,3-beta-glucan biosynthesis protein, putative ACLA_009040	*Aspergillus clavatus*	A1C9R6	517	34
	Catalytic activity: UDP-glucose+((1)) An17g02120	*Aspergillus niger* str CBS 513.88/FGSC A1513	A2R9P2	525	33
	Putative uncharacterized protein ATEG_09455	*Aspergillus terreus* str NIH 2624	Q0CA29	531	32
	1,3-beta-glucan biosynthesis protein, putative AFUB_053320	*Aspergillus fumigatus* A1163	B0Y387	515	32
	Glucan synthesis regulatory protein gs-1	*Neurospora crassa*	P38678	532	30

* References are taken from Uniprot database (http://www.uniprot.org/) for bacterial proteins and from Génolevures (http://www.genolevures.org/) for yeast proteins

** Sequence identity is measured on total query sequence length.

a *KLLA0E15862g* corresponds to the new Génolevures annotation *KLLA0E15775g*.

A second HGT candidate also found in *K. lactis*, *KLLA0C09218g*, encodes a 173 amino-acid long protein and is located in a 6 anchor point synteny block (Figure 3B). Applying the same methodology, we found sequence similarity of its product with proteins from diverse bacterial genomes, including *Xanthomonas axonopodis*, *X. campestris*, *Oceanicaulis alexandrii* and *Sphingomonas wittichii* (Table 1 and alignment in Supplementary Figure S1B). Two of these proteins are annotated as putative glyoxalases/dioxygenases. Again, a glyoxalase I gene exists in *S. cerevisiae* (*YML004C*) and has homologs in other hemiascomycetous yeasts (including *K. lactis* KLLA0F06226p protein), but such genes are 2-fold longer in sequences, and show limited similarity with our HGT candidate (average 19%, Table 1). The phylogenetic tree reconstructed shows that the *KLLA0C09218g* gene product is closer to bacterial proteins than to the yeast glyoxalase I proteins (Figure 3F), suggesting another acquisition by HGT in the *K. lactis* lineage. The function of this gene remains to be determined.

In *K. thermotolerans*, one single HGT candidate, *KLTH0E10032g*, encoding a 299 amino-acid long protein, was found in a conserved synteny block made of 12 anchor points (Figure 3C). Its product shares sequence similarity with proteins from the myxobacterium *Sorangium cellulosum* (36% identity, 51% similarity, Table 1 and alignment in Supplementary Figure S1C), the cyanobacterium *Microscilla marina* and the aquatic gamma-proteobacterium *Reinekea sp.*, the latter being annotated as a putative NADH-ubiquinone oxido-reductase. Lower similarity levels are also found with other cyanobacterial proteins (with an average of 22% pairwise identity), and with flavin reductases from mammals (from *M. musculus*, *R. norvegicus*, *E. caballus*, *B. taurus*, *C. familiaris* and *H. sapiens*, with an average of 14% identity, not shown). The limited conservation of *KLTH0E10032g* gene product with bacterial proteins (except *Sorangium cellulosum*) raises the question of its origin. If it actually originates from bacteria through HGT, it is possible that we are missing the actual donor group (it might not be represented in Uniprot database) or that its sequence has rapidly diverged after transfer. The low bootstrap values associated with the tree are consistent with the second hypothesis (Figure 3G).

Our last single HGT candidate was found in *S. kluyveri*, *SAKL0H22528g*. It encodes a 190 amino-acid long protein, located in a conserved synteny block made of 11 adjacent anchor points (Figure 3D). Its translation product shares similarity with bacterial proteins from *Burkholderia cenocepacia* (38% identity, Table 1 and alignment in Supplementary Figure S1D), *Acaryochloris marina*, *Cyanothece sp.* and *Microscilla marina* (average of 31% identity). The *B. cenocepacia* protein, even if 3-fold longer, is particularly well aligned in its C-terminal part with our HGT candidate sequence. Interestingly, the *SAKL0H22528g* product also shares similarity with a predicted protein of the *Funariaceae* moss *Physcomitrella patens* (38% pairwise identity, Table 1 and alignment in Supplementary Figure S1D), not explicitly annotated. Our HGT candidate, as the latter protein, aligns only with the C-terminal part of MoeA domain protein from *B. cenocepacia* (Supplementary Figure S1D), overlapping the SMI1 domain involved in cell wall synthesis. This could be explained by an independent HGT from the pathogenic *B. cenocepacia* to the moss [29]. No significant similarity was found with any yeast protein, but weakly similar proteins involved in glucan synthesis exist in *Neurospora crassa* and *Aspergillus* species. They show however poorly aligned sequences with our HGT candidate. All hemiascomycetous yeast proteins involved in glucan synthesis or its regulation are 3- to 10-fold longer in sequences, and show very poor alignments with SAKL0H22528p (not shown). The reconstructed phylogenetic tree shows that SAKL0H22528p is more closely related to bacterial proteins than to proteins known to act in glucan synthesis in the *Pezizomycotina* (Figure 3H), and suggests that it is a good candidate for horizontal transfer, although its original function still has to be refined.

### Duplicated horizontally acquired genes

**Figure 4:**
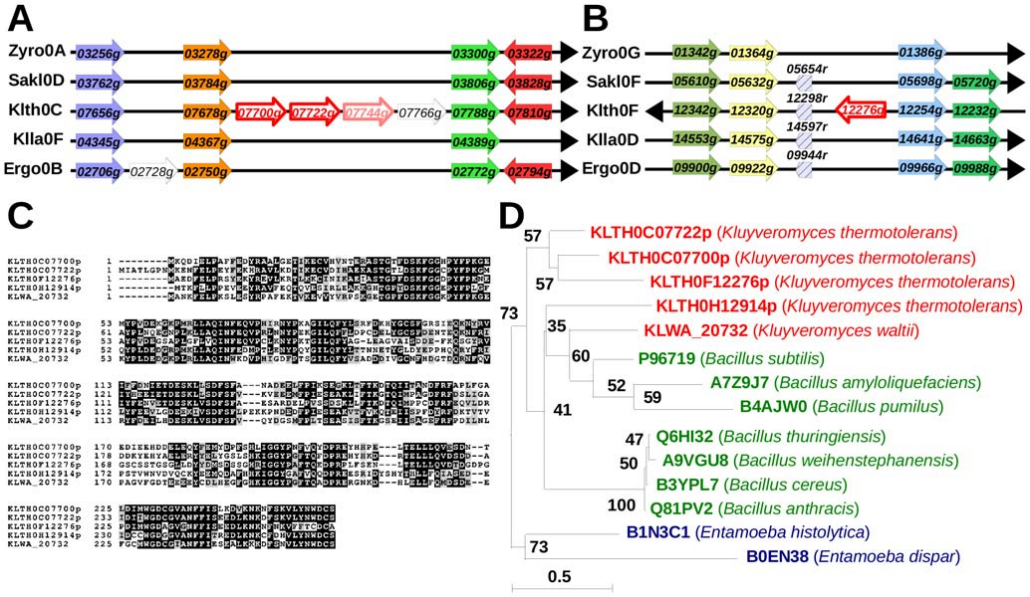
Duplicated HGT candidates in *K. thermotolerans*. (A) Part of the conserved synteny block surrounding *KLTH0C07700g* and *KLTH0C07722g* tandem genes. Same legend as Figure 3A. (B) Part of the conserved synteny block surrounding *KLTH0F12276g* gene. Same legend as Figure 3A. (C) Sequence alignment of proteins from *K. thermotolerans* and *K. waltii*. (D) Phylogenetic tree reconstructed from sequence alignment of 100 sites of *K. thermotolerans* protein family members, *K. waltii* gene, bacterial (green) and amoebal (blue) proteins. Bootstrap values are indicated next to the nodes and branch length scale is shown at bottom left.

Interestingly, we also found horizontally acquired genes present in several copies in yeast genomes, forming families of paralogs. One such case is observed in *K. thermotolerans*, where *KLTH0C07700g* and *KLTH0C07722g* genes form a pair of tandem paralogs, encoding proteins of *ca.* 260 amino-acids, inserted in a conserved synteny block made of 12 anchor points (Figure 4A). Note that four genes are inserted in this interval, and that the product of *KLTH0C07744g* gene shows weak similarity to the products of the tandem pair (not shown), and may represent a diverged repeat of the same tandem array. Two other paralogs to these genes exist on other chromosomes of *K. thermotolerans*. *KLTH0F012276g* is contained in another conserved synteny block made of 7 anchor points (Figure 4B). *KLTH0H12914g* is not in a conserved synteny block. All four genes form a species-specific protein family among our yeasts (Génolevures family GL3R3887) that shares 38 to 48% identity with proteins from various *Bacillus* species, annotated as YwqG (Table 2 and Figure 4C). These proteins also share similarity with proteins of two *Entamoeba* species, a parasitic genus in which diverse prokaryotic gene transfers have already been described [30]. The d_N_/d_S_ ratio calculated on aligned sequences of this family (Materials and Methods) shows strong functional pressure on these genes (with average d_N_/d_S_ values of 0.23, Supplementary Table S2), indicating that they are expressed in yeast and submitted to selection. Interestingly, a 259 amino-acid long protein predicted from the genome of *K. waltii* (KLWA_20732), a close relative to *K. thermotolerans*, belongs to this family. This suggests that the transfer from bacteria occurred in the ancestor of these two yeast species. The K. waltii gene is not syntenic to any of the four genes of K. thermotolerans, suggesting transfer to ectopic location or duplications followed by gene loss. The presence of large intergenes in both species, opposite to the HGT genes where corresponding regions are aligned (Supplementary Figure S2) suggest that the duplications arose before the speciation of the two species, and were subsequently lost during evolution. However, the possibility of independent transfer of the same bacterial gene to *K. thermotolerans* and *K. waltii* cannot be formally excluded.

**Table 2:** Pairwise sequence identity between *K. thermotolerans* protein family, *K. waltii* protein and best bacterial and amoebal hits.

				Identity (%)**				
Génolevures/Uniprot annotation	Species	Reference*	Length (aa)	KLTH0C07700g	KLTH0C07722g	KLTH0F12276g	KLTH0H12914g	KLWA_20732
KLTH0C07700p	*K. thermotolerans*	-	259	-	-	-	-	-
KLTH0C07722p	*K. thermotolerans*	-	267	64	-	-	-	-
KLTH0F12276p	*K. thermotolerans*	-	259	52	53	-	-	-
KLTH0H12914p	*K. thermotolerans*	-	264	48	50	44	-	-
KLWA_20732^a^	*K. waltii*	-	259	52	51	43	48	-
YwqG	*B. subtilis*	P96719	261	49	48	41	47	52
YwqG	*B. amyloliquefaciens* str FZB42	A7Z9J7	262	44	46	43	47	48
YwqG	*B. pumilus* ATCC 7061	B4AJW0	271	46	46	41	44	45
Putative uncharacterized protein BT9727_2468	*B. thuringiensis subsp konkukian*	Q6HI32	271	47	46	37	41	45
Putative uncharacterized protein BAS2507	*B. anthracis*	Q81PV2	271	48	45	37	41	44
Putative uncharacterized protein BcerKBAB4_2451	*B. weihenstephanensis* str KBAB4	A9VGU8	271	44	45	36	40	42
YwqG	*B. cereus* AH1134	B5UWG9	271	44	42	35	38	43
Putative uncharacterized protein EDI_035210	*E. dispar* SAW760	B0EN38	284	35	35	36	34	32
Putative uncharacterized protein EHI_001040	*E. histolytica* HM-1:IMSS	B1N3C1	267	30	31	30	31	29

* References are taken from Uniprot database (URL: http://www.uniprot.org/) for bacterial and Entamoeba proteins and from Génolevures (URL: http://www.genolevures.org/) for yeast proteins.

** Sequence identity is measured on total query sequence length.

a *K. waltii* gene KLWA_20732 sequence was taken from annotation published in Kellis *et al.* (2004).

**Figure 5:**
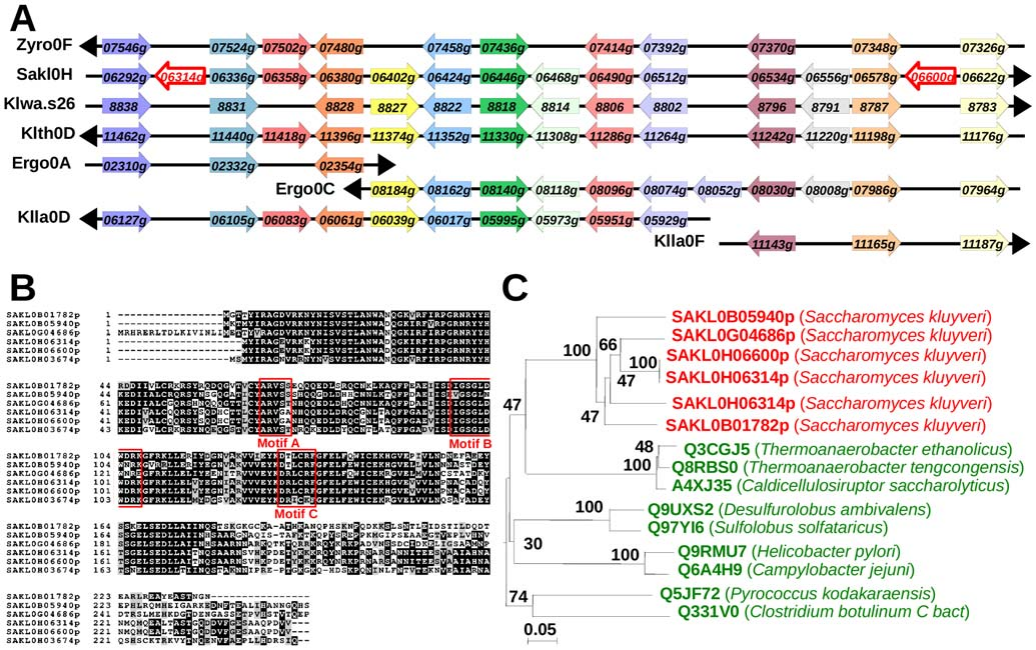
Duplicated HGT candidates in *S. kluyveri*. (A) Chromosomal region surrounding *SAKL0H06314g* and *SAKL0H06600g* and conserved syntenic regions of the other protoploid genomes including *K. waltii*. Same legend as Figure 3A. (B) Sequence alignment of the six putative horizontally transferred serine recombinases of *S. kluyveri*. Red rectangles indicate the conserved motifs (A, B and C) within the catalytic domain according to Grindley *et al.* [33]. (C) Phylogenetic tree built from protein alignment of the six *S. kluyveri* proteins and of the closest serine recombinase from archaea (Q9UXS2, Q97YI6 and Q5JF72) and bacteria especially Firmicutes (Q3CGJ5, Q8RBS0, A4XJ35 and Q331V0) and proteobacteria (Q9RMU7 and Q6A4H9). After horizontal gene transfer, the first putative serine recombinase in *S. kluyveri* has been successively duplicated in the genome. The presence of identical genes (*SAKL0H06314g* and *SAKL0H06600g*) suggests a recent duplication event or the result of a conversion event. Bootstrap values are indicated next to the nodes.

Another case of amplification of horizontally acquired genes was found in *S. kluyveri*. Three genes, *SAKL0B01782g*, *SAKL0G04686g* and *SAKL0H06600g*, detected according to our method (Figure 5A) are members of a family also including *SAKL0B05940g, SAKL0H03674g* and *SAKL0H06314g*. The latter gene falls in the same synteny block as *SAKL0H06600g* (Figure 5A), but was not originally detected in our method because of a synteny breakpoint in the *K. lactis* genome (not shown in Figure 5A). The predicted gene products of this family share 56 to 100% amino-acid identity between themselves (Table 3 and Figure 5B). Interestingly, the 100% identity between *SAKL0H06314g* and *SAKL0H06600g*, separated by 12 other genes along the same chromosome, extends to 113 and 138 nt in promoter and terminator regions, respectively, indicating a recent duplication event. The three genes not identified from our method are located in conserved synteny blocks restricted to fewer species including those of the *Lachancea* clade (Figure 5A and Supplementary Figure 3). The products of these six genes have no significant similarity in eukaryotes so far, thus they form a species-specific protein family in *S. kluyveri* (Génolevures family GL3R3665). Instead, they share similarity with putative serine recombinases of the IS*607* family found in bacteria and archaea (Table 3). The most similar ones (27–31% identity) are in *Thermoanaerobacter ethanolicus*, *T. tengcongensis* and *Caldicellulosiruptor saccharolyticus*, three species of Firmicutes (Table 3 and Figure 5C). These proteins are composed of a DNA binding domain in their N-terminal part, that is highly conserved in all six *S. kluyveri* proteins (first 50 amino-acids, Figure 5B) and a catalytic domain covers almost the rest of their sequence [31], [32]. Motifs A, B and C of the catalytic domain of the serine recombinases [33] are conserved in *S. kluyveri* proteins with, however, two exceptions: the catalytic serine, at position 71 in motif A (Figure 5B), is replaced by a glycine in SAKL0H06314p and SAKL0H06600p, which should abolish the activity of these proteins, and the Arg-132 in motif C is replaced by a threonine residue in SAKL0B01782p and SAKL0B05940p. The d_N_/d_S_ ratio between members of the six gene family shows a bias towards synonymous against non-synonymous mutations (d_N_/d_S_ values of 0.19 on average, Supplementary Table S2), suggesting a functional selective pressure on these genes. Because the serine recombinases are part of mobile elements in prokaryotes, we looked for the presence/absence of the 6 HGT genes in different strains of *S. kluyveri* isolated from various parts of the world (Supplementary Table S3) using PCR primers in the two flanking genes. Thus, we did not observe any polymorphism for five out of the six HGT genes in all studied strains. Indeed, PCR results for the *SAKL0H03674g* gene suggests that this locus is highly polymorphic, as revealed by the absence of amplicon in five strains, the presence of two amplicons in two other strains (CBS6626 and CBS10368), and of an insertion in another strain (CBS10369, Supplementary Table S4). Note that this chromosomal region is particularly rearranged (Supplementary Figure S3C). Nevertheless, the transposase activity of this protein family in yeast remains to be demonstrated.

**Table 3:** Pairwise sequence identity between *K. kluyveri* protein family and best bacterial and archaeal hits.

				Identity (%)**					
Génolevures/Uniprot annotation	Species	Reference*	Length (aa)	SAKL0B01782p	SAKL0B05940p	SAKL0H03674p	SAKL0H06314p	SAKL0H06600p	SAKL0G04686p
SAKL0B01782p	*S. kluyveri*	-	237	-	-	-	-	-	-
SAKL0B05940p	*S. kluyveri*	-	253	59	-	-	-	-	-
SAKL0H03674p	*S. kluyveri*	-	249	57	56	-	-	-	-
SAKL0H06314p	*S. kluyveri*	-	248	56	57	61	-	-	-
SAKL0H06600p	*S. kluyveri*	-	248	56	57	61	100	-	-
SAKL0G04686p	*S. kluyveri*	-	254	56	62	57	62	62	-
Regulatory protein, MerR:Resolvase	*T. ethanolicus*	Q3CGJ5	197	29	28	27	27	27	29
Predicted site-specific integrase-resolvase	*T. tengcongensis*	Q8RBS0	196	29	28	27	27	27	28
Resolvase, N-terminal domain	*C. saccharolyticus*	A4XJ35	201	29	28	28	28	28	29
Putative resolvase	*D. ambivalens*	Q9UXS2	194	25	24	24	24	24	24
First ORF in transposon ISC1904	*S. solfataricus*	Q97YI6	192	25	23	23	24	24	24
Putative transposase OrfA	*H. pylori*	Q9RMU7	217	26	24	24	25	25	24
Putative uncharacterized protein OrfA	*C. jejuni*	Q6A4H9	212	26	23	22	23	23	23
Predicted site-specific integrase/resolvase	*P. kodakaraensis*	Q5JF72	209	27	23	24	22	22	25
Putative IS transposase (OrfA)	*C. botulinum* C bacteriophage	Q331V0	219	24	25	25	23	23	26

* References are taken from Uniprot database (http://www.uniprot.org/) for bacteria and archae and from Génolevures (http://www.genolevures.org/) for yeast proteins.

** Sequence identity is measured on total query sequence length.

## Discussion

Cases of horizontal gene transfers have been previously reported in hemiascomycetous yeast genomes. Here, we have exploited the remarkable synteny conservation among five distantly related yeast species to systematically screen for the presence of species-specific insertion of genes. Using this strategy, we identified 15 novel genes of HGT origin (11 intervening genes and four additional family members), representing a minimum of 6 independent transfer events that occurred in 3 distinct species. This increases the number of previously published cases of HGT in yeasts by 50%. As of today, HGT genes have been found in almost all yeast species (except *Z. rouxii*) in which they were sought for, suggesting that this mechanism is more frequent than usually imagined. Given the restrictive method used in this work, it is likely that other cases of HGT were missed either because they did not fall into conserved synteny blocks or because they were ancestral to several lineages and, therefore, not retained as “species-specific” genes. As an illustration of this, the *URA1* gene, previously shown to have been acquired by an ancestor of *S. cerevisiae* and *S. kluyveri* [22], has syntenic orthologs in *K. thermotolerans* and *K. lactis*, that were, therefore, considered as anchor points in our synteny blocks and not as intervening genes. We also identified highly similar orthologs of *S. cerevisiae* HGT genes *YOL164W* and *YJL217W* in *K. thermotolerans*, suggesting more ancestral HGT events. Another reason limiting the discovery of HGT genes is the present content of databases. The collection of 244 species-specific intervening genes remaining without homology in databases is puzzling (see Supplementary Table S1). It is possible that some of them correspond to HGT from non-sequenced group of organisms. With the development of high-throughput sequencing technologies providing new sequences of environmental or non-cultivated species, one can hope that the number of trans-kingdom homologs will increase.

From the total number of HGT identified today among Hemiascomycetes (Figure 1), yeasts follow the amoeba where up to 152 HGT candidates were found in *Trichomonas vaginalis* [4], but are far ahead of *Metazoa* where only a few HGT events were described so far (*e.g.* in the Nematode, ICL and MS genes subsequently fused [34], and in *Ciona intestinalis*, cellulose synthase presumed to have been transferred in the early Urochordates ancestor [35]).

Our results extend the idea that HGT genes can rapidly duplicate in their novel host. One case of duplicated HGT gene was previously reported in *K. lactis*, and two cases in *Y. lipolytica*, but were not analyzed further [20]. We show here that duplications occurred in *K. thermotolerans* and *S. kluyveri*, forming families of up to six genes. Although the latter case concerns a bacterial transposase, it is unlikely that its duplication in yeast results from its activity because no polymorphism is observed among the tested strains, and two copies have a mutation in the catalytic domain. Outside yeasts, other cases of duplicated horizontally acquired genes were described in amoeba [36], [37]. Such duplications suggest that HGT genes are functional in their host and submitted to selective pressures as judged from low d_N_/d_S_ ratios. Similar figures are also found for the previously published cases of duplicated HGT in hemiascomycetous yeasts (respectively d_N_/d_S_ values of 0.03 and 0.13 on average for *K. lactis* and *Y. lipolytica,* Supplementary Table S2).

As judged from database annotations, horizontally acquired genes of yeasts correspond to a large variety of functions, primarily concerning cellular metabolism (Figure 1). In one previously reported example, the successive integrations of HGT genes of the biotin biosynthesis pathway (*BIO3* and *BIO4*) from diverse bacterial origins into *S. cerevisiae* genome argue for the reconstruction of a previously lost function [23].

The transfer of genes from bacteria to yeasts raises questions about the mechanism involved in foreign DNA uptake and integration into chromosomes. Trans-kingdom conjugation has been observed between *E. coli* and *S. cerevisiae* cells [38]. Bacterial conjugation, however, involves long DNA segments, while we always observe single-gene insertions. Transformation of yeast cells by exogenous DNA is an other possibility. In the laboratory, specific treatments are needed to increase the frequency of transformation to a measurable level. But very rare events can play important role within large populations and long evolutionary time scales. Fragments of mitochondrial DNA can integrate chromosomes at double-strand breaks (DSB) [39] and several such fragments (NUMTs) are present in yeast genomes [40]. It is possible that HGT would be similarly facilitated by chromosomal DSBs. Remarkably, however, NUMTs are essentially found outside or at the border of conserved synteny blocks. The fact that horizontally acquired genes tend to be smaller than the average yeast genes (median of 248±96 codons compared to 410±11, Figure 1) is consistent with transformation by short DNA fragments. Two exceptions nevertheless exist, *BDS1* in *S. cerevisiae* (647 amino-acids) and *YdhR* in *D. hansenii* (776 amino-acids). Cases of introgression of large DNA fragments have been mentioned among the *Saccharomyces sensu stricto* complex [41], [42], and even from more distantly related yeasts, *e.g. Zygosaccharomyces bailii* into *S. cerevisiae* [43]. In addition, some beer strains are hybrids between *S. cerevisiae* and *S. kudriavzevii* [44], [45], in which introgressed chromosomal fragments can be exchanged. Cases of horizontal gene transfers between yeasts have also been identified through phylogenetic incongruence of the gene tree (*e.g.* the DAL5 transporter family in [46]).

Finally, as our screening method tolerated small local rearrangements within conserved synteny blocks (Materials and Methods), we are able to examine whether insertion of HGT genes is accompanied or not by other local rearrangements. An intriguing contiguity between the HGT gene *KLLA0B05269g* and a segmental duplication with chromosome D is observed in *K. lactis*, but we cannot decide whether the two events are concomitant or not. We also observe the presence of tRNA genes next to HGT genes (Figures 3A,D, 4B, S2A,C,D and S3B,C), sometimes associated with relics of transposable elements (Long-Terminal Repeats, LTRs, Supplementary Figures S2C,D and Figures S3 B,C). Other cases of intervening genes are also observed in the vicinity (Figures 3D, 4A, S2A,B and S3C,D), suggesting that these regions may be more susceptible to rearrangements, possibly because they are more susceptible to meiotic double-strand breaks than others, as previously reported in *S. cerevisiae* chromosome III [47], [48].

## Materials and Methods

### Protoploid yeast genomes

Sequences and annotations used in this work were taken from the Génolevures website (http://www.genolevures.org/), and published by Dietrich *et al.* (*Eremothecium* (*Ashbya*) *gossypii* genome) [26], Dujon *et al.* (*Kluyveromyces lactis* genome) [20] and the Génolevures Consortium (*Zygosaccharomyces rouxii*, *Saccharomyces* (*Lachancea*) *kluyveri* and *Kluyveromyces* (*Lachancea*) *thermotolerans* genomes) [13]. The new genera *Lachancea* has been introduced by Kurtzmann (2003) [49]. The *Kluyveromyces waltii* genome used for some comparisons has been annotated by Kellis *et al.* [50]. Protein families taken from Génolevures website were previously defined from systematic comparisons of complete predicted proteomes from nine hemiascomycetous species [51]. Orthologs for the five protoploid *Saccharomycetaceae* were identified from protein families using gene neighborhood conservation [52].

### Synteny block construction

Construction of synteny blocks conserved within the genomes of *Z. rouxii*, *S. kluyveri*, *K. thermotolerans*, *K. lactis* and *E. gossypii* is based on the physical adjacency of sets of orthologous genes along chromosomes, controlled by two parameters: the minimum number of orthologous genes common to all 5 species, used as anchor points, and the maximum number of tolerated non-orthologous genes between two adjacent anchor points. Adjacency was deduced from sequence-derived chromosome maps as annotated by the Génolevures Consortium (http://www.genolevures.org/). Note that tandem gene repeats are considered as equivalent to a single gene by our method. We set the two parameters to 5 minimum anchor points and 25 maximum intervening genes, by extension of a previous work [13]. Note that only 8 cases of more than 10 consecutive intervening genes were actually found within synteny blocks, with a maximum of 18 intervening genes in a raw. Finally, we used annotated tRNA genes to consolidate existing synteny blocks (annotated using tRNAscan [53] for *K. waltii* draft genome).

### Identification of putative HGT

Within synteny blocks, we extracted as “intervening genes” for further analysis only those that are present in one species and absent in all four others. For all intervening genes, we checked for the possible presence of homologs at ectopic location in other yeast species. Remaining species-specific intervening genes were finally compared to the NR nucleotide database of NCBI (release 10.5, ftp://ftp.ncbi.nih.gov) using Blastx tool [54], without filter of low complexity sequence, and with the default threshold of e-value of 10. Best hits were extracted for each gene, eliminating the gene itself. This list was finally filtered manually to find significant hits with proteins belonging to any other species but yeasts, by applying a threshold of 1.0E^−6^ to the e-value and a minimum of 15% identity with compared sequence.

### HGT characterization

Sequences were aligned using MAFFT program [55], alignments were curated using Gblocks tool (version 0.91b) [56], removing gaps and saturated positions and thus keeping only informative sites. Identity percentages were calculated over the total query length. Phylogenetic trees were inferred from sequence divergence, using PHYML tool with a JTT substitution model corrected for heterogeneity among sites by a gamma-law distribution using 4 categories of substitution rates, proportion of invariable sites and the alpha parameter of the gamma-law distribution optimized according to the data (version 3.0) [57], validated by 100 aLRT replicates [58]. The resulting trees were drawn using Treedyn [59] or NJplot [60] programs. ClustalW [61] tool was used for global alignment of single HGT candidates with their best bacterial hits. Gblocks, PHYML and Treedyn programs are those of Phylogeny.fr web server [62].

### Calculation of d_N_/d_S_ ratio

We used the number of non-synonymous over synonymous mutations as a measure of sequence divergence of paralogous copies of HGT family members. The program yn00 of the PAML package [63] has been used with default parameters. This program detects non-synonymous and synonymous sites (respectively N and S) within a protein family, and then counts for each protein pair the number of non-synonymous mutations by non-synonymous sites and synonymous mutations by synonymous site (respectively d*_N_* and d*_S_*).

### Experimental data

PCR amplifications were performed to detect the presence/absence of the six putative serine recombinase genes in the genomes of the different *S. kluyveri* strains (Supplementary Table S3). Ploidy of the strains is taken from [27]. Primers were designed in the two flanking genes and synthesized by Eurogentec (Seraing, Belgium). Their characteristics are listed in Supplementary Table S5. Reactions were performed in a final volume of 25 µl in an Applied Biosystems thermocycler (Courtaboeuf, France) using *ex-Taq* DNA polymerase from Takara (France) in the recommended buffer and about 50 ng of genomic DNA as a template. The following conditions were used: an initial denaturation of 2 min at 94°C followed by 30 cycles of denaturation at 94°C for 30 s, annealing at 56°C for 30 s, and elongation at 72°C for 2 min, and a final elongation at 72°C for 2 min. PCR products were electrophoresed on 1% agarose gel, migrated in TAE 1X (*ca.* 100V), colored with BET and visualized through UV.

## Supporting Information

Figure S1 Alignments of single HGT candidates in K. lactis, K. thermotolerans and S. kluyveri. These alignments have been produced using ClustalW tool (Materials and Methods). (A) Sequence alignment of KLLA0B05269p with bacterial proteins. Aeromonas salmonicida protein is annotated as hypothetical protein, and Bacillus cereus protein as member of the SMI1/KNR4 family. (B) Sequence alignment of KLLA0C09218p with bacterial proteins. Xanthomonas axonopodis and Oceanicaulis alexandrii proteins are annotated as hypothetical, Xanthomonas campestris protein as putative glyoxalase/bleomycin resistance protein/dioxygenase, and Sphingomonas wittichii protein as glyoxalase/bleomycin resistance protein/dioxygenase. The N-terminal extension in our candidate sequence with respect to bacterial sequences goes beyond a conserved in-frame methionine, and could be explained by an alternative upstream start codon or an incorrect annotation. (C) Sequence alignment of KLTH0E10032p with the Sorangium cellulosum protein, annotated as hypothetical. The sequences share 36% identity, as measured on total length of query protein. (D) Sequence alignment of SAKL0H22528p with bacterial and moss proteins. Physcomitrella patens protein is predicted, and Burkholderia cenocepacia strain MC0-3 protein is annotated as MoeA domain protein.
(0.54 MB PPT)

Figure S2 Part of conserved syntenic regions surrounding HGT candidates in K. thermotolerans and K. waltii. Gaps in syntenic chromosomal regions supports a possible loss of genes. (A) Syntenic region of K. waltii HGT candidate (KLWA_20732) in K. thermotolerans and S. kluyveri. Orthologous genes, inferred from sequence similarity for K. waltii, are colored, intervening genes are white, and tRNA genes are indicated by short hatched arrows. Arrows represent gene orientation. Note that the scale is not respected. We observe a well conserved synteny between all three species of the Lachancea clade, with a large intergenic region at the location corresponding to KLWA_20732 in K. thermotolerans, but not in S. kluyveri. (B) Syntenic region of K. thermotolerans tandem pair HGT candidates in K. waltii and S. kluyveri. Same legend as (A). Grey gene KLWA_23011 has an ectopic homolog. Note that KLTH0C07744g shows poor similarity with the genes of the tandem pair, and thus may also represent a diverged tandem repeat. Here, we observe a very large intergenic region at the location corresponding to the tandem pair in K. waltii, not found in S. kluyveri. (C) Syntenic region of K. thermotolerans KLTH0F12276g HGT candidate in K. waltii and S. kluyveri. Same legend as (A). Note that this region is syntenic with the four other protoploid species (Figure 4B). Remarkably, we observe a synteny breakpoint in K. waltii. This region involves a tRNA proline gene in K. thermotolerans and S. kluyveri, and another tRNA glycine in K. waltii, identified using tRNAscan (Material and Methods). (D) Syntenic region of K. thermotolerans KLTH0H12914g HGT candidate in K. waltii and S. kluyveri. Same legend as (A). Here again, we observe large intergenic regions in K. thermotolerans and K. waltii. Interestingly, this region is even larger in S. kluyveri, and contains two tRNA genes and remnants from transposable elements (Long-Terminal Repeats, LTRs).
(0.13 MB PPT)

Figure S3 Part of conserved synteny regions surrounding the putative serine recombinase genes in S. kluyveri (SAKL0H06314g and SAKL0H06600g are represented in Figure 5). (A) SAKL0B05940g was not initially identified as an intervening gene due to the synteny breakpoint occurring in Ergo0F/Ergo0B precisely at the locus corresponding to the serine recombinase insertion. (B) SAKL0G04686g was detected as an intervening gene despite the fact that the region is highly rearranged. A large sequence inversion is present in both Klth0G and Klwa.s47 (black hatched rectangle), compared to Sakl0G, ZYRO0D, ERGO0A and KLLA0A (the flanking genes have been inverted). The orange pentagon at the inversion border represents a conserved tRNA gene identified using tRNAscan (Materials and Methods). The yellow pentagon (ERGO0A07744r) represents a non-coding tRNA gene whose localization is specific to E. gossypii. Black arrows in rectangles symbolize LTR or relics of LTR. (C) SAKL0H03674g was not initially identified as an intervening gene due to the synteny breakpoint in Klla0E that separated the syntenic region into two distant regions of this chromosome. The blue pentagon represents a tRNA gene conserved in all species. It is interesting to note that the synteny breakpoint region is also highly rearranged as in (B) with the presence of LTRs. (D) SAKL0B01782g was detected as an intervening gene in a synteny block made of 9 anchor points, five of them being represented on this figure. White arrows correspond to distinct intervening genes.
(0.18 MB PPT)

Table S1 Set of 244 species-specific intervening genes which have no significant hit in NR database.
(0.21 MB DOC)

Table S2 Measures of dN/dS ratio for duplicated HGT genes.
(0.12 MB DOC)

Table S3 Yeast strains used in this work.
(0.12 MB DOC)

Table S4 Results of PCR amplification of the six serine recombinase genes in S. kluyveri strains.
(0.12 MB DOC)

Table S5 Primers used to amplify the serine recombinase genes.
(0.11 MB DOC)
