# Supplementary figures and images for: Insertion of Horizontally Transferred Genes within Conserved Syntenic Regions of Yeast Genomes
Source: PLoS One. 2009 Aug 5;4(8):e6515. doi: 10.1371/journal.pone.0006515 (PMC2715888; doi:10.1371/journal.pone.0006515)

## Slide 1
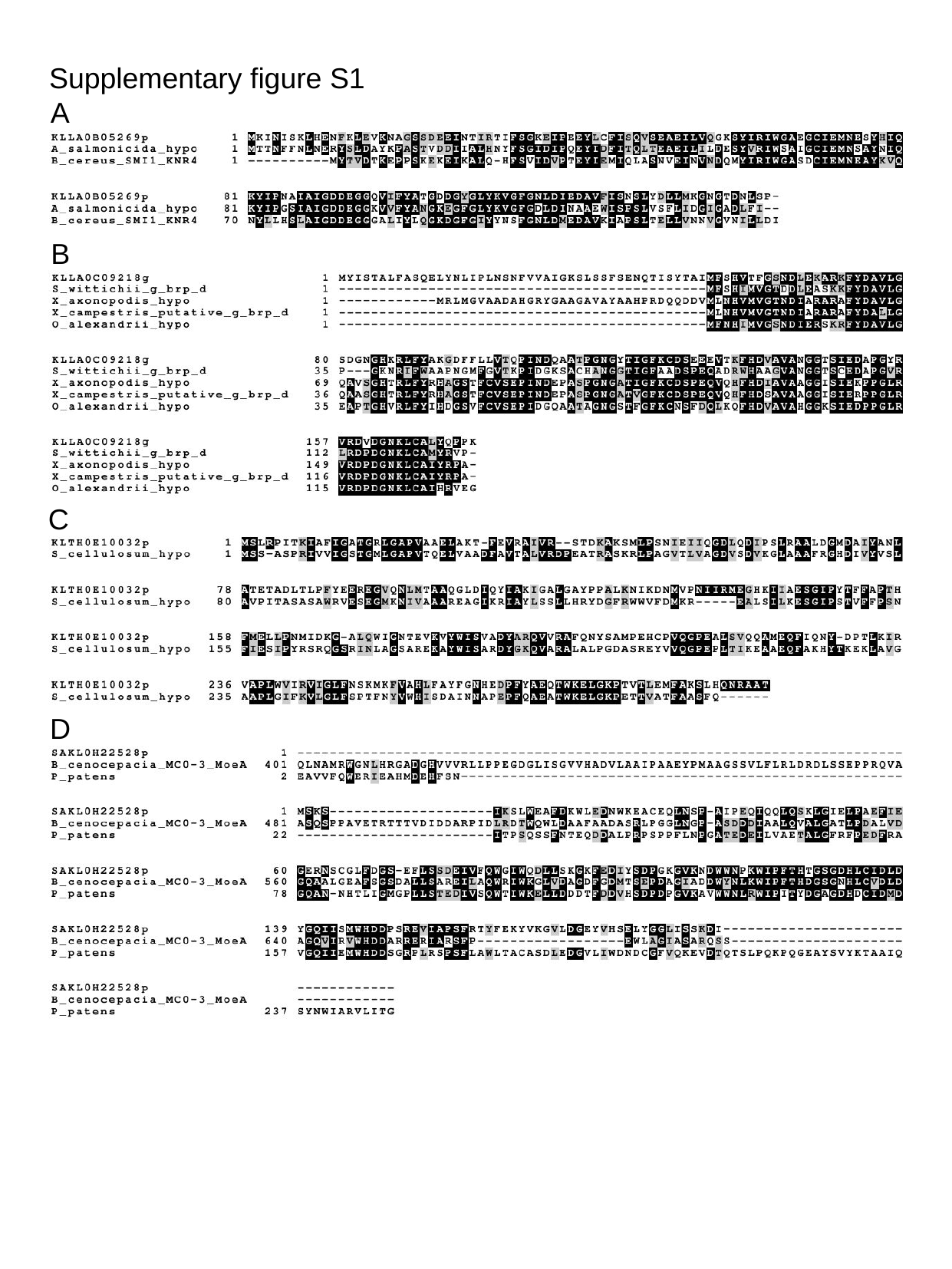

Supplementary figure S1
A
B
C
D

Supplement: Figure S1 — Alignments of single HGT candidates in K. lactis, K. thermotolerans and S. kluyveri. These alignments have been produced using ClustalW tool (Materials and Methods). (A) Sequence alignment of KLLA0B05269p with bacterial proteins. Aeromonas salmonicida protein is annotated as hypothetical protein, and Bacillus cereus protein as member of the SMI1/KNR4 family. (B) Sequence alignment of KLLA0C09218p with bacterial proteins. Xanthomonas axonopodis and Oceanicaulis alexandrii proteins are annotated as hypothetical, Xanthomonas campestris protein as putative glyoxalase/bleomycin resistance protein/dioxygenase, and Sphingomonas wittichii protein as glyoxalase/bleomycin resistance protein/dioxygenase. The N-terminal extension in our candidate sequence with respect to bacterial sequences goes beyond a conserved in-frame methionine, and could be explained by an alternative upstream start codon or an incorrect annotation. (C) Sequence alignment of KLTH0E10032p with the Sorangium cellulosum protein, annotated as hypothetical. The sequences share 36% identity, as measured on total length of query protein. (D) Sequence alignment of SAKL0H22528p with bacterial and moss proteins. Physcomitrella patens protein is predicted, and Burkholderia cenocepacia strain MC0-3 protein is annotated as MoeA domain protein. (0.54 MB PPT) [file pone.0006515.s001.ppt]
