# Supplementary material for: Insertion of Horizontally Transferred Genes within Conserved Syntenic Regions of Yeast Genomes
Source: PLoS One. 2009 Aug 5;4(8):e6515. doi: 10.1371/journal.pone.0006515 (PMC2715888; doi:10.1371/journal.pone.0006515)
Supplement: Figure S2 — Part of conserved syntenic regions surrounding HGT candidates in K. thermotolerans and K. waltii. Gaps in syntenic chromosomal regions supports a possible loss of genes. (A) Syntenic region of K. waltii HGT candidate (KLWA_20732) in K. thermotolerans and S. kluyveri. Orthologous genes, inferred from sequence similarity for K. waltii, are colored, intervening genes are white, and tRNA genes are indicated by short hatched arrows. Arrows represent gene orientation. Note that the scale is not respected. We observe a well conserved synteny between all three species of the Lachancea clade, with a large intergenic region at the location corresponding to KLWA_20732 in K. thermotolerans, but not in S. kluyveri. (B) Syntenic region of K. thermotolerans tandem pair HGT candidates in K. waltii and S. kluyveri. Same legend as (A). Grey gene KLWA_23011 has an ectopic homolog. Note that KLTH0C07744g shows poor similarity with the genes of the tandem pair, and thus may also represent a diverged tandem repeat. Here, we observe a very large intergenic region at the location corresponding to the tandem pair in K. waltii, not found in S. kluyveri. (C) Syntenic region of K. thermotolerans KLTH0F12276g HGT candidate in K. waltii and S. kluyveri. Same legend as (A). Note that this region is syntenic with the four other protoploid species (Figure 4B). Remarkably, we observe a synteny breakpoint in K. waltii. This region involves a tRNA proline gene in K. thermotolerans and S. kluyveri, and another tRNA glycine in K. waltii, identified using tRNAscan (Material and Methods). (D) Syntenic region of K. thermotolerans KLTH0H12914g HGT candidate in K. waltii and S. kluyveri. Same legend as (A). Here again, we observe large intergenic regions in K. thermotolerans and K. waltii. Interestingly, this region is even larger in S. kluyveri, and contains two tRNA genes and remnants from transposable elements (Long-Terminal Repeats, LTRs). (0.13 MB PPT) [file pone.0006515.s002.ppt]

## Slide 1
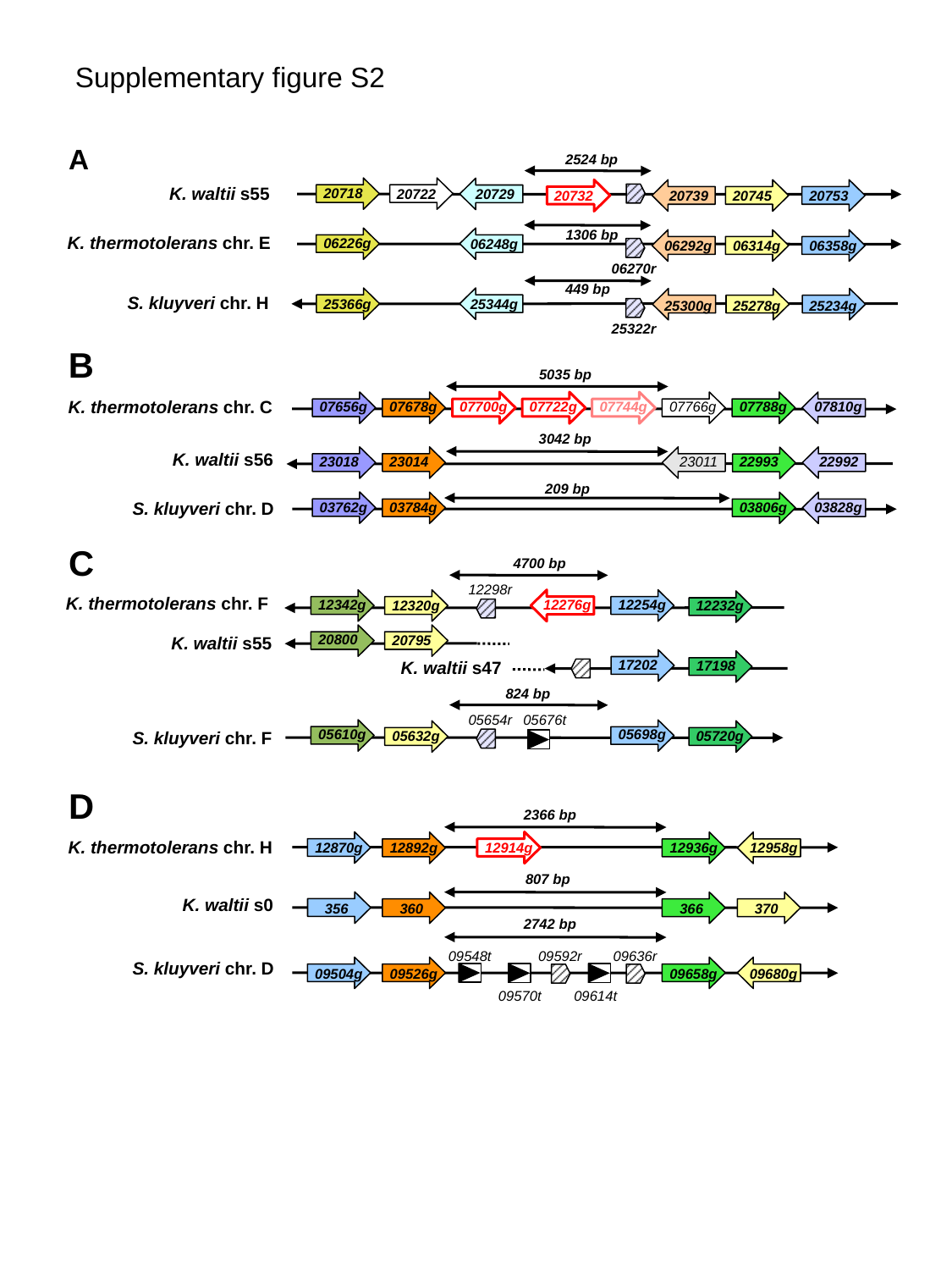

Supplementary figure S2
A
2524 bp
K. waltii s55
20718
20722
20729
20732
20739
20745
20753
1306 bp
K. thermotolerans chr. E
06226g
06248g
06292g
06314g
06358g
06270r
449 bp
S. kluyveri chr. H
25366g
25344g
25300g
25278g
25234g
25322r
B
5035 bp
K. thermotolerans chr. C
07656g
07678g
07700g
07722g
07744g
07766g
07788g
07810g
3042 bp
K. waltii s56
23018
23014
22993
23011
22992
209 bp
03762g
03784g
03806g
03828g
S. kluyveri chr. D
C
4700 bp
12298r
K. thermotolerans chr. F
12342g
12276g
12254g
12320g
12232g
20800
20795
K. waltii s55
17202
17198
K. waltii s47
824 bp
05654r
05676t
05610g
05698g
05632g
05720g
S. kluyveri chr. F
D
2366 bp
K. thermotolerans chr. H
12870g
12892g
12914g
12936g
12958g
807 bp
K. waltii s0
356
360
366
370
2742 bp
09548t
09592r
09636r
S. kluyveri chr. D
09504g
09526g
09658g
09680g
09570t
09614t
