# Supplementary material for: Insertion of Horizontally Transferred Genes within Conserved Syntenic Regions of Yeast Genomes
Source: PLoS One. 2009 Aug 5;4(8):e6515. doi: 10.1371/journal.pone.0006515 (PMC2715888; doi:10.1371/journal.pone.0006515)
Supplement: Figure S3 — Part of conserved synteny regions surrounding the putative serine recombinase genes in S. kluyveri (SAKL0H06314g and SAKL0H06600g are represented in Figure 5). (A) SAKL0B05940g was not initially identified as an intervening gene due to the synteny breakpoint occurring in Ergo0F/Ergo0B precisely at the locus corresponding to the serine recombinase insertion. (B) SAKL0G04686g was detected as an intervening gene despite the fact that the region is highly rearranged. A large sequence inversion is present in both Klth0G and Klwa.s47 (black hatched rectangle), compared to Sakl0G, ZYRO0D, ERGO0A and KLLA0A (the flanking genes have been inverted). The orange pentagon at the inversion border represents a conserved tRNA gene identified using tRNAscan (Materials and Methods). The yellow pentagon (ERGO0A07744r) represents a non-coding tRNA gene whose localization is specific to E. gossypii. Black arrows in rectangles symbolize LTR or relics of LTR. (C) SAKL0H03674g was not initially identified as an intervening gene due to the synteny breakpoint in Klla0E that separated the syntenic region into two distant regions of this chromosome. The blue pentagon represents a tRNA gene conserved in all species. It is interesting to note that the synteny breakpoint region is also highly rearranged as in (B) with the presence of LTRs. (D) SAKL0B01782g was detected as an intervening gene in a synteny block made of 9 anchor points, five of them being represented on this figure. White arrows correspond to distinct intervening genes. (0.18 MB PPT) [file pone.0006515.s003.ppt]

## Slide 1
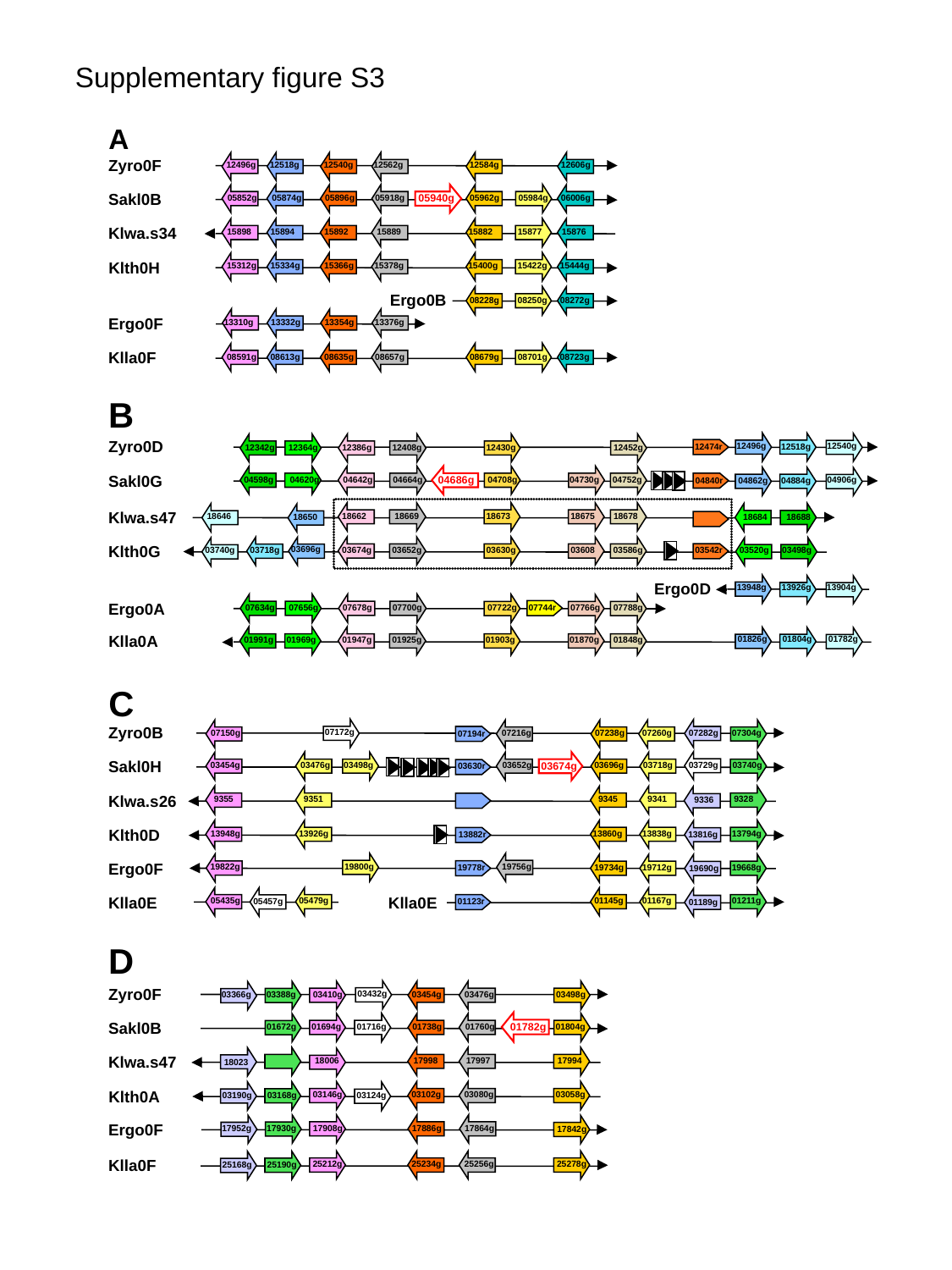

Supplementary figure S3
A
Zyro0F
12496g
12518g
12540g
12562g
12584g
12606g
Sakl0B
05940g
05852g
05874g
05896g
05918g
05962g
05984g
06006g
Klwa.s34
15898
15894
15892
15889
15882
15877
15876
Klth0H
15312g
15334g
15366g
15378g
15400g
15422g
15444g
Ergo0B
08228g
08250g
08272g
Ergo0F
13310g
13332g
13354g
13376g
Klla0F
08591g
08613g
08635g
08657g
08679g
08701g
08723g
B
Zyro0D
12496g
12540g
12518g
12474r
12342g
12364g
12386g
12408g
12430g
12452g
Sakl0G
04686g
04598g
04620g
04642g
04664g
04708g
04730g
04752g
04906g
04840r
04862g
04884g
Klwa.s47
18662
18669
18673
18675
18678
18646
18684
18688
18650
Klth0G
03696g
03718g
03740g
03520g
03498g
03674g
03652g
03630g
03608
03586g
03542r
Ergo0D
13926g
13948g
13904g
Ergo0A
07634g
07656g
07678g
07700g
07722g
07766g
07788g
07744r
Klla0A
01804g
01826g
01782g
01991g
01969g
01947g
01925g
01903g
01870g
01848g
C
Zyro0B
07172g
07304g
07150g
07216g
07238g
07260g
07282g
07194r
Sakl0H
03674g
03454g
03476g
03498g
03652g
03696g
03718g
03729g
03740g
03630r
Klwa.s26
9355
9351
9345
9341
9328
9336
Klth0D
13948g
13926g
13860g
13838g
13794g
13816g
13882r
Ergo0F
19822g
19800g
19756g
19778r
19734g
19712g
19668g
19690g
Klla0E
Klla0E
05435g
05479g
01145g
01167g
01211g
05457g
01123r
01189g
D
Zyro0F
03432g
03410g
03454g
03476g
03498g
03388g
03366g
Sakl0B
01782g
01694g
01716g
01738g
01760g
01804g
01672g
Klwa.s47
18006
17998
17997
17994
18023
Klth0A
03146g
03102g
03080g
03058g
03168g
03190g
03124g
Ergo0F
17908g
17886g
17864g
17930g
17952g
17842g
Klla0F
25212g
25234g
25256g
25278g
25190g
25168g
