# Supplementary material for: Insertion of Horizontally Transferred Genes within Conserved Syntenic Regions of Yeast Genomes
Source: PLoS One. 2009 Aug 5;4(8):e6515. doi: 10.1371/journal.pone.0006515 (PMC2715888; doi:10.1371/journal.pone.0006515)
Supplement: Table S1 — Set of 244 species-specific intervening genes which have no significant hit in NR database. (0.21 MB DOC) [file pone.0006515.s004.doc]

**Supplementary table S1.** Set of 244 species-specific intervening genes which have no significant hit in NR database.
