# Supplementary material for: Insertion of Horizontally Transferred Genes within Conserved Syntenic Regions of Yeast Genomes
Source: PLoS One. 2009 Aug 5;4(8):e6515. doi: 10.1371/journal.pone.0006515 (PMC2715888; doi:10.1371/journal.pone.0006515)
Supplement: Table S2 — Measures of dN/dS ratio for duplicated HGT genes. (0.12 MB DOC) [file pone.0006515.s005.doc]

**Supplementary table S2.** Measures of d_N_/d_S_ ratio for duplicated HGT genes.
