# Supplementary material for: Insertion of Horizontally Transferred Genes within Conserved Syntenic Regions of Yeast Genomes
Source: PLoS One. 2009 Aug 5;4(8):e6515. doi: 10.1371/journal.pone.0006515 (PMC2715888; doi:10.1371/journal.pone.0006515)
Supplement: Table S4 — Results of PCR amplification of the six serine recombinase genes in S. kluyveri strains. (0.12 MB DOC) [file pone.0006515.s007.doc]

**Supplementary table S4.** Results of PCR amplification of the six serine recombinase genes in *S. kluyveri* strains.
