# Supplementary material for: Insertion of Horizontally Transferred Genes within Conserved Syntenic Regions of Yeast Genomes
Source: PLoS One. 2009 Aug 5;4(8):e6515. doi: 10.1371/journal.pone.0006515 (PMC2715888; doi:10.1371/journal.pone.0006515)
Supplement: Table S5 — Primers used to amplify the serine recombinase genes. (0.11 MB DOC) [file pone.0006515.s008.doc]

**Supplementary table S5.** Primers used to amplify the serine recombinase genes.
